# Supplementary material for: Active acetylcholine receptors prevent the atrophy of skeletal muscles and favor reinnervation
Source: Nat Commun. 2020 Feb 26;11:1073. doi: 10.1038/s41467-019-14063-8 (PMC7044284; doi:10.1038/s41467-019-14063-8)
Supplement: Supplementary file 2 — Supplementary Information [file 41467_2019_14063_MOESM2_ESM.pdf]

Active acetylcholine receptors prevent the atrophy of skeletal muscles and favor reinnervation

Cisterna et al.

Supplementary Figures

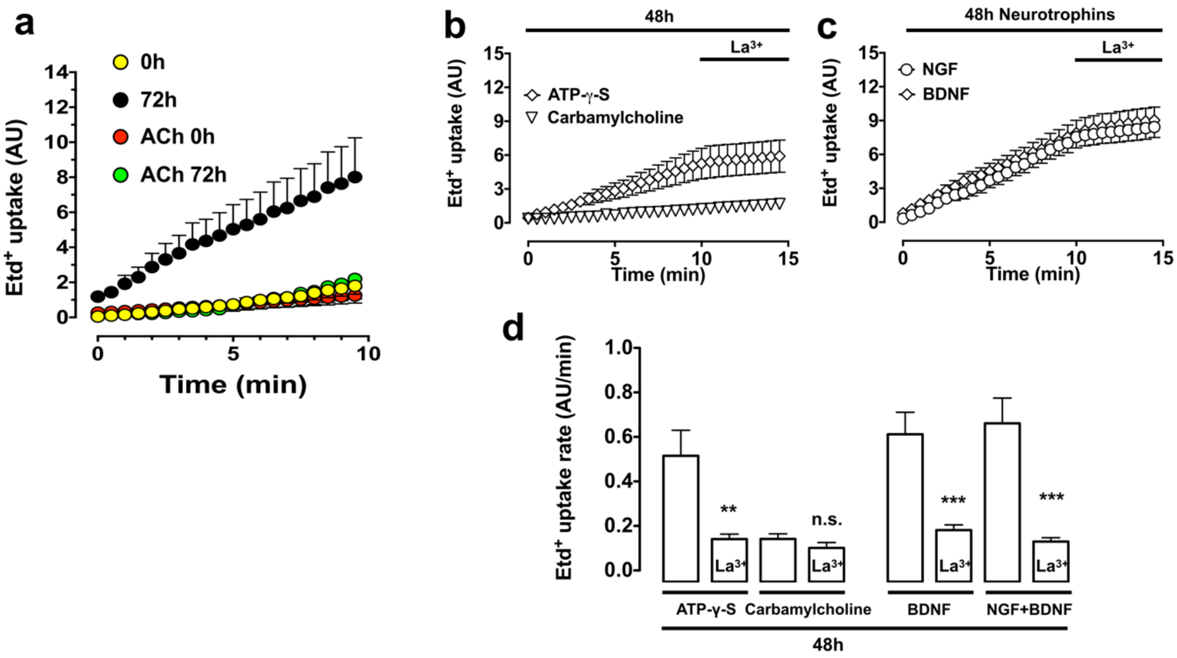

Supplementary Figure 1. **Acetylcholine (ACh) or Methyl-carbamylcholine (Cmc), but not adenosine triphosphate (ATP) or neurotrophins prevent the increase in sarcolemmal permeability in cultured skeletal myofibers**. In primary cultures of myofibers from *flexor digitorum brevis* muscles of $Cx43^{fl/fl}Cx45^{fl/fl}$ mice the Etd^+ uptake was measured in real time at 0 and 72 h of culture in control conditions or 0 and 72 h in presence of 500 nM ACh (**a**), or at 48 h of culture in the presence of 500 μM ATP-γ-S, or 200 nM Cmc (**b**), or 50 ng/ml NGF or 50 ng/ml BDNF (**c**). Each fiber was recorded under basal conditions for 10 min and then 200 μM La^{3+} was added and recorded for 5 min. **d**, Etd^+ uptake rate in myofibers. N=4; at least five myofibers recorded in each animal, each value is the mean ± SEM. n.s.: non-significant difference. ** $p < 0.005$ and *** $p < 0.001$, for effect of La^{3+} compared with basal conditions by Student's *t* test.

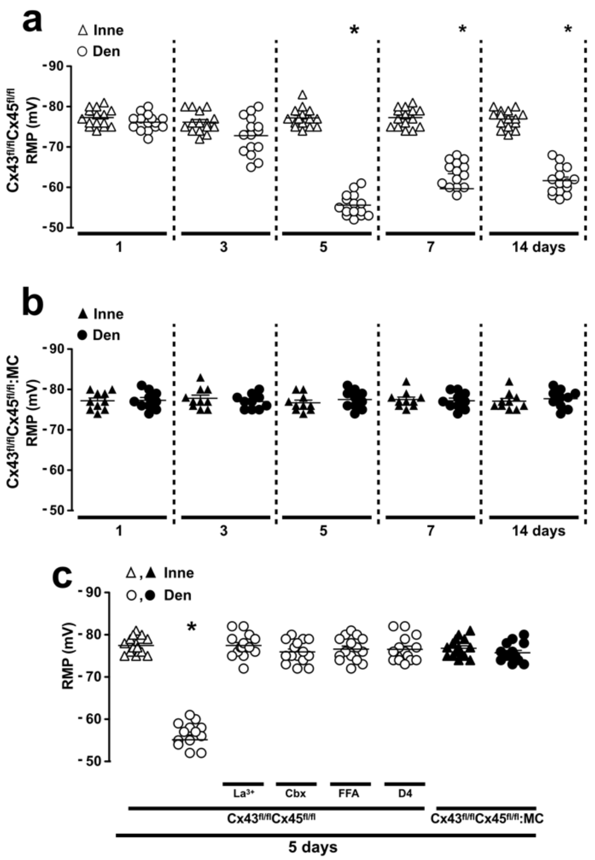

Supplementary Figure 2. **Lack of expression of Cx43 and Cx45 prevents and acute inhibition of Cx HCs reverts the reduction in resting membrane potential (RMP) of denervated skeletal myofibers**. **a**, Unilateral sciatic nerve transections were performed in $Cx43^{fl/fl}Cx45^{fl/fl}$ mice (white). **b**, in $Cx43^{fl/fl}Cx45^{fl/fl}$:MC mice (black). At days 1, 3, 5, 7 and 14 post-denervation, the *in vivo* RMP was evaluated in *flexor digitorum brevis* muscles in innervated (Inne; triangles) and denervated (Den; circles) myofibers. **c**, At day 5, the RMP was measured followed by treatment for 90 min with hemichannel blockers as indicated (200 µM La^{3+}, 100 µM Cbx, 300 µM AFF or 200 nM D4). N=5; at least twenty myofibers recorded in each animal. * $p < 0.05$, for denervated myofibers compared with innervated myofibers by ANOVA with Bonferroni *post hoc* test.

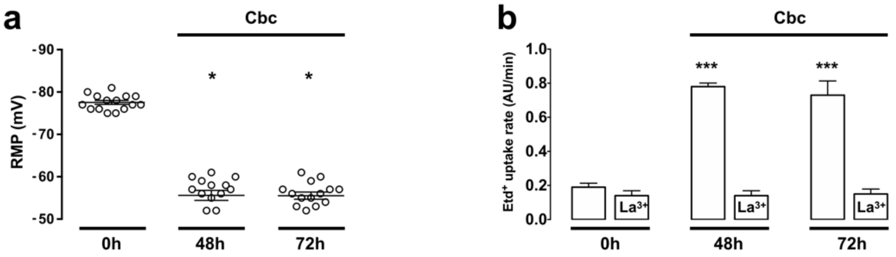

Supplementary Figure 3. **Carbachol (Cbc) does not reverse the increase in sarcolemmal permeability or the reduction in resting membrane potential (RMP) in cultured skeletal myofibers**. Primary myofibers of *flexor digitorum brevis* muscles of $Cx43^{fl/fl}Cx45^{fl/fl}$ mice were cultured for 0 h, 48 h, and 72 h. Cbc was added to the culture from 48 h and on. RMP (**a**), and Etd^{+} uptake (**b**) were measured; each fiber was recorded under basal conditions for 10 min and then 200 µM La^{3+} was added and recording was continued for an additional 5 min. RMP was recorded for at least twenty myofibers in each of 5 independent experiments. * $p < 0.05$, for 48 h or 72 h compared with 0 h of culture by ANOVA with a Bonferroni *post hoc* test. Etd^{+} uptake was recorded for at least five myofibers in each of 4 independent experiments (N=4); each value is the mean ± SEM. *** $p < 0.001$, for effect of La^{3+} compared with basal conditions by the Student's *t* test.

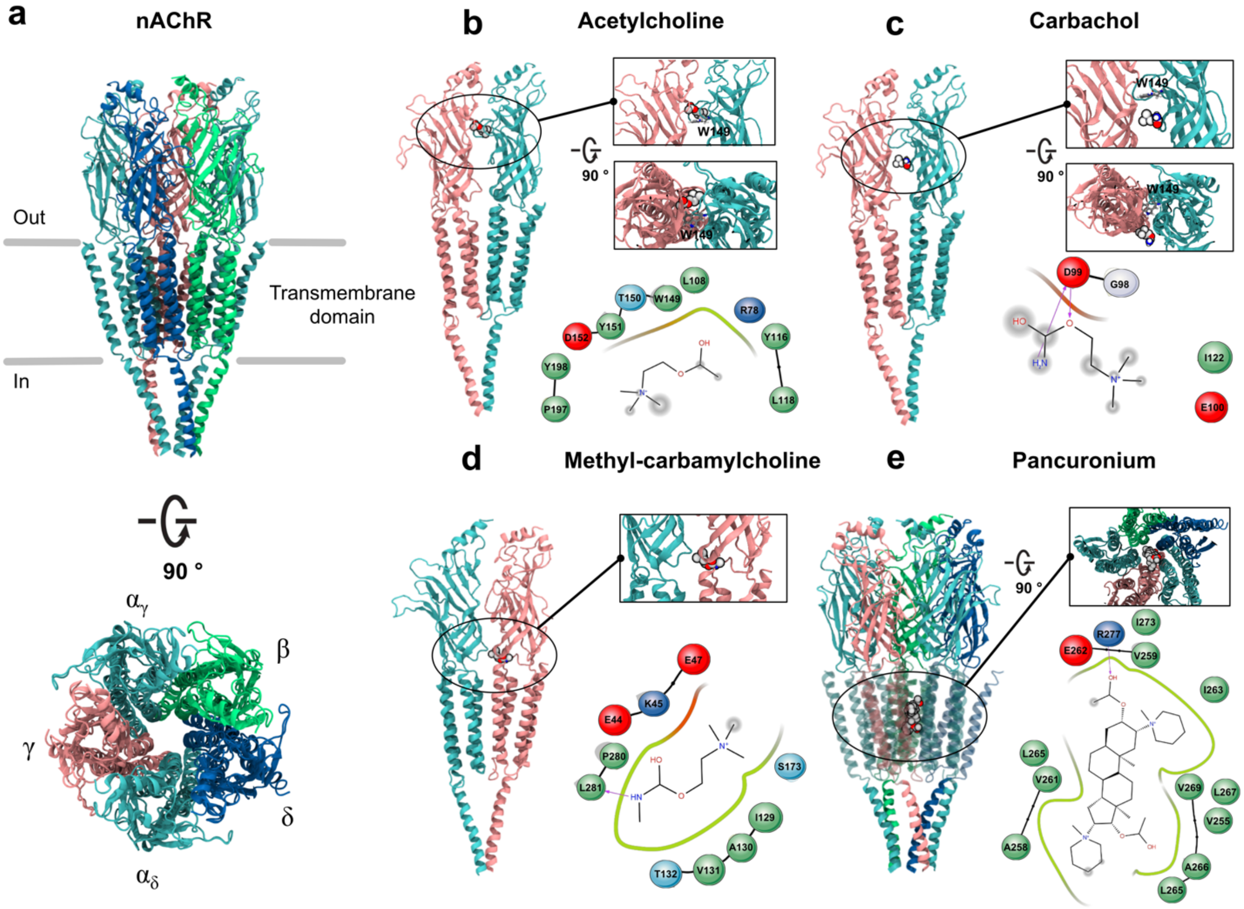

Supplementary Figure 4. **Predicted binding conformation of blockers and activators inside nicotinic acetylcholine receptor**. **a**, General representation of nicotinic acetylcholine receptor (nAChR) (PDB ID:2BG9; *Torpedo* nAChR). A side view of the nAChR depicts the three domains: intracellular, extracellular and transmembrane. A top view of the receptor shows the αγ and αδ subunits in cyan, γ in pink, β in green, δ in blue. **b**, 3D (side and top view) and 2D representation of acetylcholine (ACh) interacting between αγ-γ subunits in the extracellular domain. ACh is stabilized by hydrophobic interactions with Y116, T150, Y198, D152, W149, Y151 and R78. **c**, 3D (side and top view) and 2D representation of carbachol (Cbc) interacting between αγ-γ subunits in the extracellular domain. The molecule is stabilized by hydrophobic interactions with E100, I122 and G98. In addition, two hydrogen bonds are formed between Cbc and D99. **d**, 3D (side and top view) and 2D representation of methyl-carbamylcholine (Cmc) interacting between γ-αδ subunits at the interface of the extracellular and transmembrane domain. Cmc is stabilized by hydrophobic interactions with A130, E47, E44, K45, T132, P280, I129, S173 and V131. In addition, a hydrogen bond interaction is established between the N-terminal carbamate portion and L281. **e**, 3D (side and top view) and 2D representation of pancuronium (Pcu) interacting with the transmembrane domain. Pcu is located in the pore of the channel and is mainly stabilized by hydrophobic interactions and a hydrogen bond formed between one hydroxyl group and R277. The ligands are represented in spheres using conventional color coding (carbon-gray; hydrogen-white; oxygen-red) and some key residues are rendered in stick mode (W149). For clarity, some subunits are hidden, depending on the complex representation. In the 2D diagram, negative, positive, polar and hydrophobic residues are represented by red, blue, cyan, and green spheres, respectively. H-bond (backbone) is depicted as a purple arrow. The binding pocket is represented by a line drawn around the ligand, colored by the color of the nearest residue. Solvent exposure is indicated on the ligand atoms, and by the break in the line drawn around the pocket.

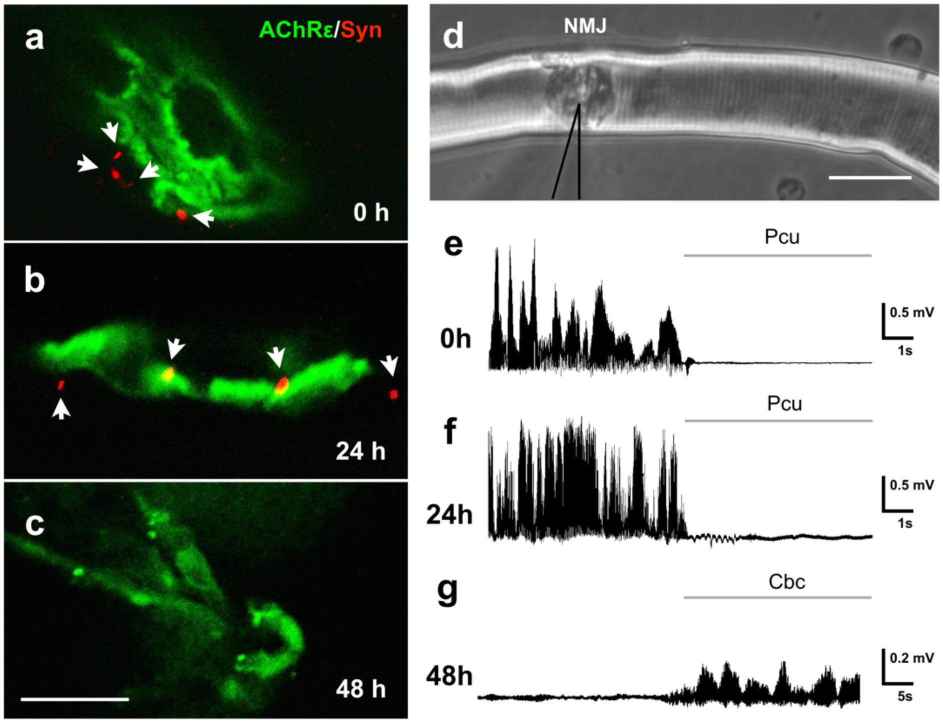

Supplementary Figure 5. **Neuromuscular junctions (NMJ) of cultured myofibers present excitatory miniature end-plate potentials (MEPPs) triggered by release of acetylcholine**. Myofibers of *flexor digitorum brevis* muscles from $Cx43^{fl/fl}Cx45^{fl/fl}$ mice were used. Immunoreactivity of acetylcholine receptor ε subunit (AChεR in green) and synaptophysin (Syn in red; arrow heads) detected by confocal immnofluorescence microscopy at 0 (**a**), 24 (**b**) and 48 h (**c**) of culture. Calibration bar: 20 µm. **d**, Contrast phase view of a myofiber showing the recording glass microelectrode located in close proximity (denoted by black lines) to NMJ for detection of excitatory MEPPs. Calibration bar: 15 µm. Recording of MEPPs under control conditions at 0h (**e**), 24h (**f**) or 48h (**g**). Pancuronium (Pcu) was used to inhibit and carbachol (Cbc) to stimulate nAChR.

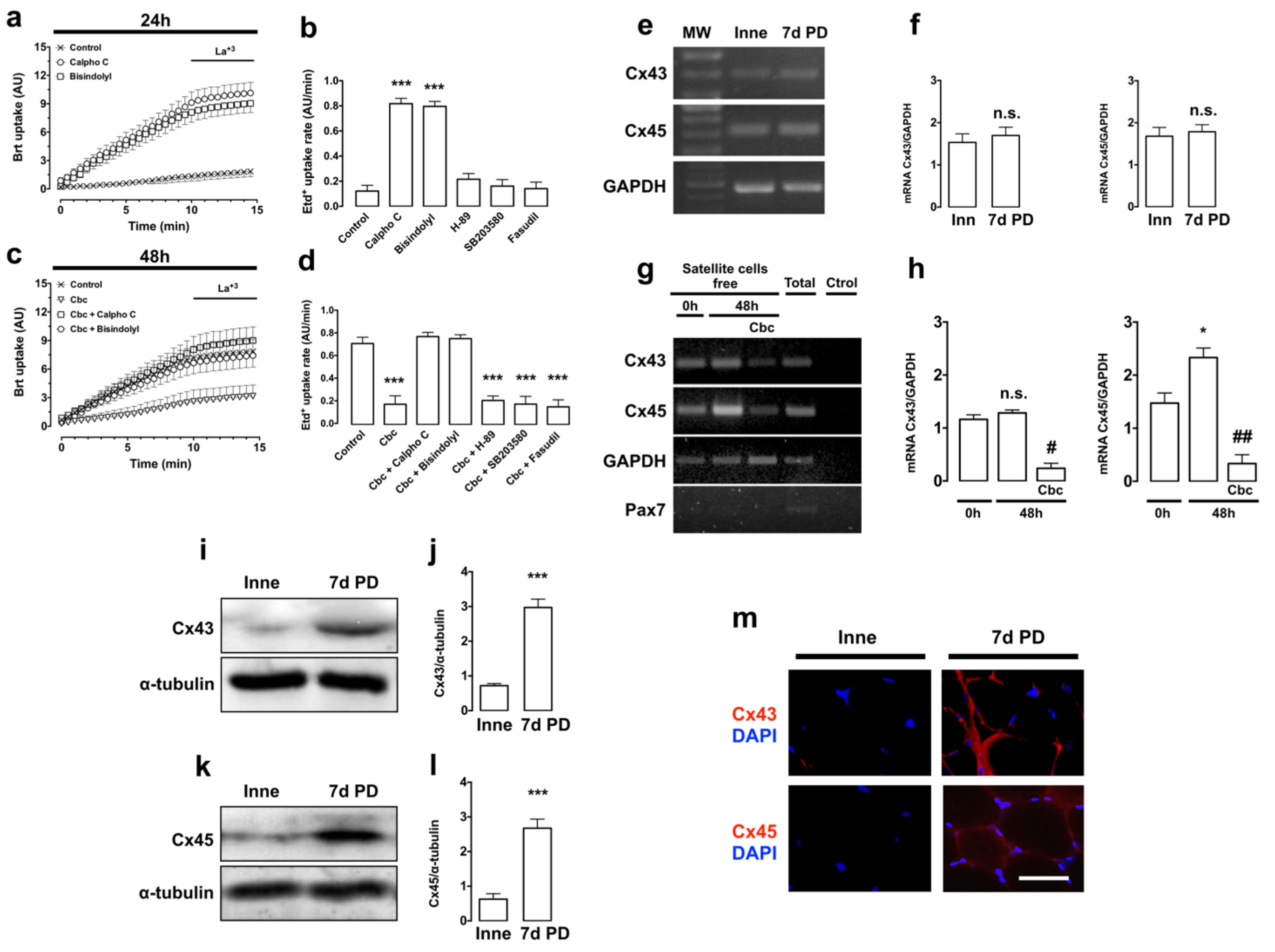

Supplementary Figure 6. **Myofibers of *flexor digitorum brevis* muscles from Cx43$^{fl/fl}$Cx45$^{fl/fl}$ mice were used**. **a** and **b**, Membrane permeability evaluated as Etd^{+} uptake in the presence of protein kinase blockers calphostin C (Calpho C), bisindolylmaleimide (Bisindolyl), H-89 dihydrochloride hydrate (H-89), SB203580 or Fasudil. **c** and **d**, Likewise, these blockers were co-incubated with Cbc+Calpho C, Cbc+Bisindolyl, Cbc+H-89, Cbc+SB203580 or Cbc+Fasudil for 24 or 48 h. N=4; seven myofibers were recorded in each independent experiment, each value is the mean ± SEM. *** $p < 0.001$. compared to control at 0 h and 48 h of culture by ANOVA with Bonferroni *post hoc* test. **e** and **f**, RT-PCR of Cx43 and Cx45 mRNAs in innervated (Inne) and 7 days post-denervation (7d PD) muscles. **g** and **h**, RT-PCR of Cx43 and Cx45 mRNAs in isolated myofibers at 0, 48, and 48 h treated with carbachol (Cbc). The levels of each connexin mRNA were normalized to the levels of GAPDH mRNA. (**i-l**) Western blot analysis of Cx43 and Cx45 in whole innervated and 7 days post-denervated muscle, (**m**) immunofluorescence of Cx43 and Cx45 in innervated and 7 days post-denervated muscle.

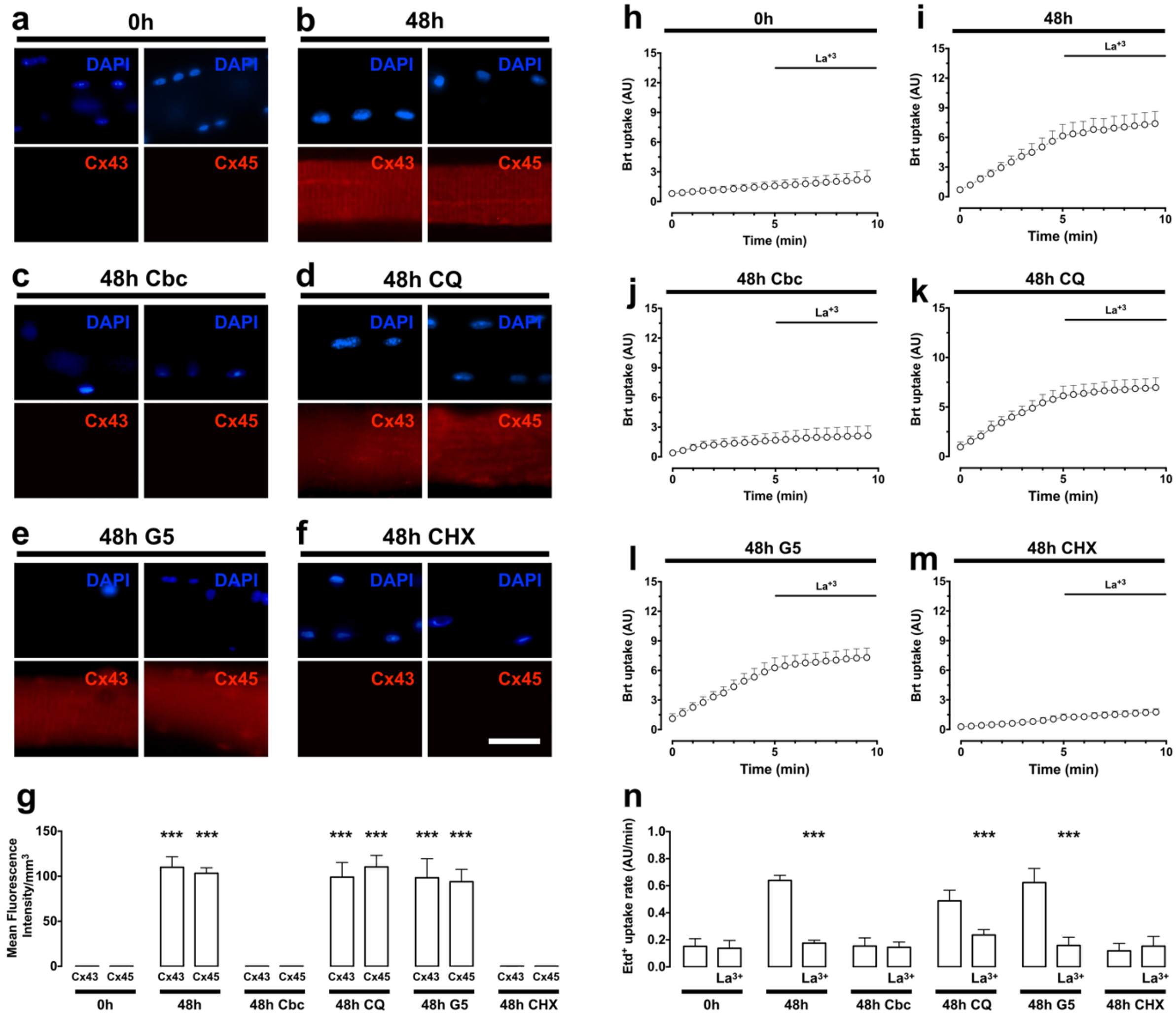

Supplementary Figure 7. **Blockade of lysosomes or 26S proteasomes does not prevent the increase in sarcolemma permeability and Cx immunoreactivity in cultured skeletal myofibers**. Myofibers of *flexor digitorum brevis* muscles from Cx43$^{fl/fl}$Cx45$^{fl/fl}$ mice were used. **a**-**f**, Cx43 and Cx45 immunofluorescence (Red: Cx43 or Cx45 immunoreactivity and blue: nuclei staining with DAPI). **g**, Quantification of fluorescence through the mean fluorescence intensity per mm^3. N=6; at least five myofibers from each independent experiment were quantified, each value corresponds to the mean ± SEM. *** $p < 0.001$. **h**-**m**, Membrane permeability evaluated as Etd$^+$ uptake at the indicated time points. Group of myofibers were treatment as follow: 200 nM carbachol (Cbc; **c** and **j**), 50 µg/ml chloroquine diphosphate salt (CQ; **d** and **k**), 1 µg/ml G5, a ubiquitin isopeptidase inhibitor I (G5; **e** and **l**), or 100 µg/ml cycloheximide (CHX; **f** and **m**). In dye uptake experiments, the first 5 min were recorded under basal conditions and the following 5 min cells were treated with 200 µM La^{3+} to block Cx HCs. **n**, Etd$^+$ uptake rate during basal conditions. N=6; at least five myofibers from each independent experiment were recorded, each value corresponds to the mean ± SEM. *** $p < 0.001$, for the effect of La^{3+} compared to basal conditions by Student's *t* test. Scale bar: 50 µm.

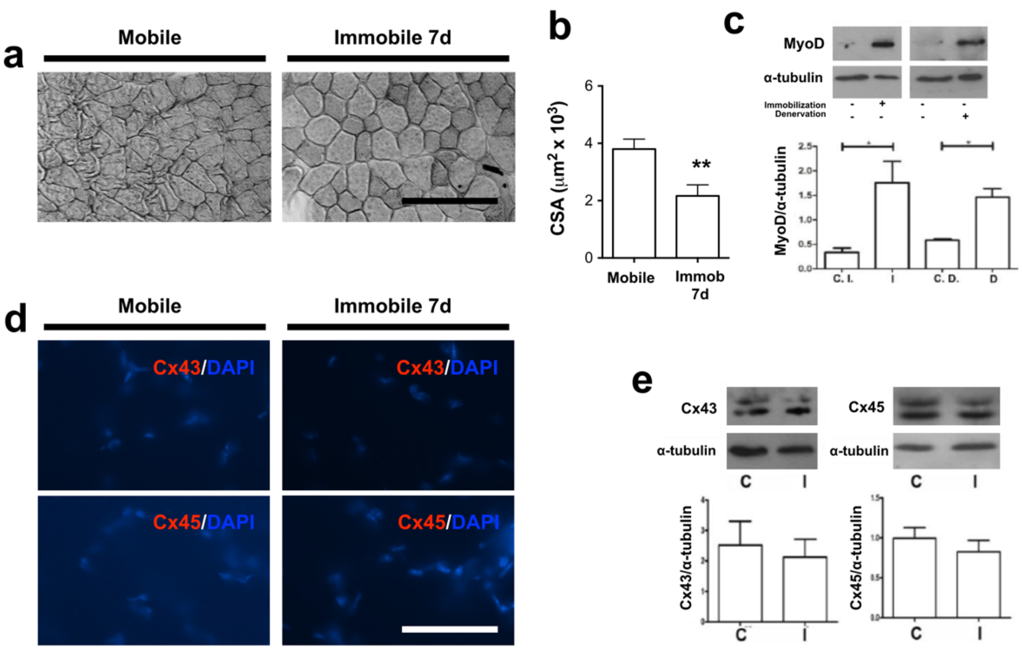

Supplementary Figure 8. **Immobilization decreases cross-sectional (CSA) area but does not increase Cx reactivity**. Unilateral Achilles tenotomy was performed in $Cx43^{fl/fl}Cx45^{fl/fl}$ mice. Gastrocnemius muscles were dissected from both limbs at 7 days post unilateral tenotomy (control: mobile and post-tenotomy: immobile 7d). **a**, Hematoxylin-eosin stained cross-sections. **b**, CSA was measured. **c**, Western blot analysis indicates a significant increase in the relative levels of MyoD protein in immobile 7d and denervated compared with their contralateral controls. N=7, each value is the mean ± SEM. ** $p < 0.005$ by Student's *t* test. **d**, immunofluorescence against Cx43 and Cx45. **e**, Western blot analysis of Cx43 and Cx45 proteins.

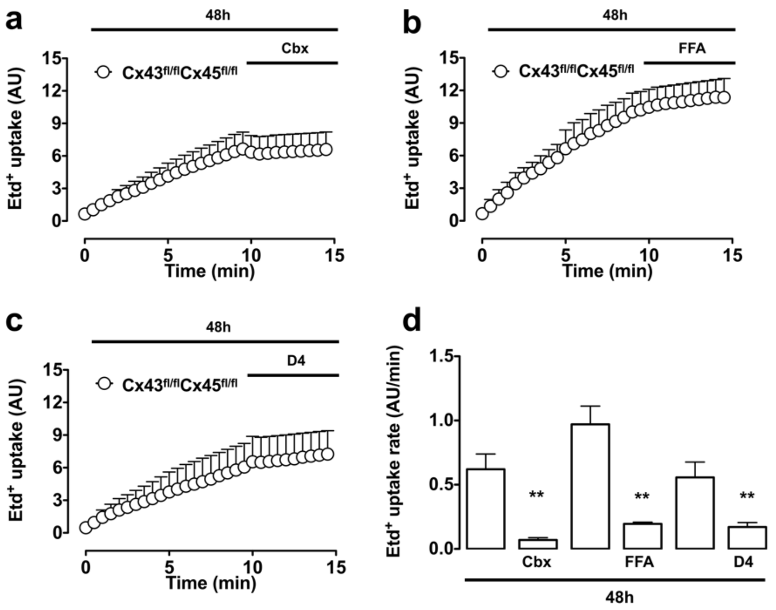

Supplementary Figure 9. Blockade of connexin hemichannels prevents the increase in sarcolemma permeability in cultured skeletal myofibers. In cultures of myofibers of *flexor digitorum brevis* muscles of $Cx43^{fl/fl}Cx45^{fl/fl}$ mice, Etd^+ uptake was measured in real time at 48 h. **a**, **b** and **c**, Etd^+ uptake was evaluated under basal conditions for 10 min of recording and then the myofibers were treated with 100 µM carbenoxolone (Cbx) (**a**), 300 µM flufenamic acid (FFA) (**b**), or 100 nM D4 (**c**). After 10 min basal recording of Etd^+ uptake myofibers were treated with 200 µM La^{3+}. **d**, Etd^+ uptake rate in myofibers. N=5; at least four myofibers recorded in each independent experiment, each value is the mean ± SEM. ** $p < 0.005$, for effect of each blocker compared with basal conditions by Student's *t* test.

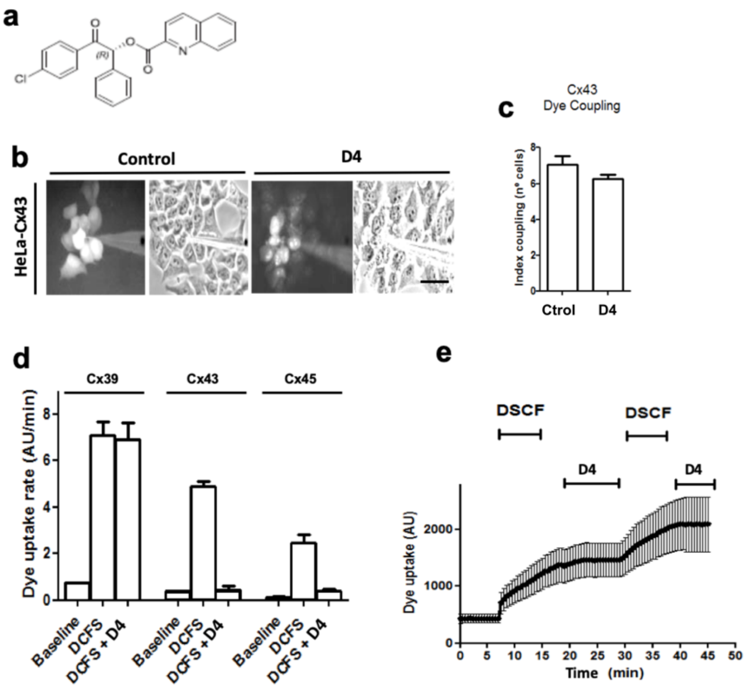

Supplementary Figure 10. D4 blocks Cx43 hemichannels but not Cx43 gap junctions. **a**, Molecular structure of (R)-2-(4-chlorophenyl)-2-oxo-1-phenylethyl quinoline-2-carboxylate ($C_{24}H_{16}ClNO_3$; MW 401.85 g.mol-1) was identified using a structure-based virtual screening campaign towards a Cx43 comparative model, and tracer transport inhibition assays in HeLa cells transfected with Cx43. The molecule is called D4, and only the R chiral form is active. **b**, The application of 200 μM D4 did not affect gap junction mediated dye coupling between HeLa cells transfected with Cx43 (HeLa-Cx43). Fluorescent panels show transfer of Lucifer yellow microinjected in one cell in HeLa cells to other cells under control conditions or after treatment for 5 min with D4. In both conditions, the microinjected dye diffused to several neighboring cells. **c**, The graph shows the index of coupling (the average number of cells to which the dye spread from the microinjected cell in successful trials), which was not affected by D4. N=5; 10 cells microinjected in each independent experiment; each value is the mean ± SEM. $p > 0.05$. **d**, Functional state of hemichannels constituted of different connexins (Cx39, Cx43 or Cx45) evaluated using the ethidium uptake measured in time-lapse experiments in control saline solution, in divalent cation-free solution (DCFS) to increase the open probability of hemichannels, and in DCFS plus 10 nM D4. Hemichannels composed of Cx43 or Cx45 but not Cx39 were blocked by D4. N=3; 20 cells recorded in each experiment; each value is the mean ± SEM. *** $p < 0.001$. **e**, a representative experiment of the fluorescence intensity in arbitrary units (AU) of ethidium bound to intracellular nucleic acid. The application of DCFS increases the slope of the dye uptake curve and the application of D4 (10 nM) in DCFS drastically reduced the dye uptake. Further description and characterization of this molecule will be published elsewhere.

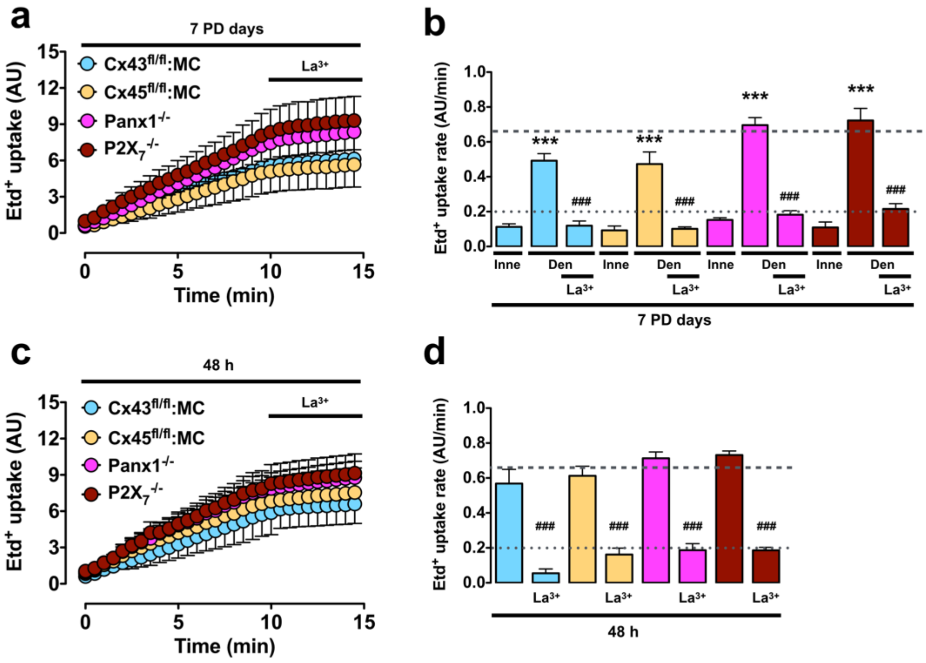

Supplementary Figure 11. Lack of Cx43, Cx45, Panx1 or $P2X_7$ does not prevent the increase in sarcolemmal permeability in cultured skeletal myofibers. Myofibers of *flexor digitorum brevis* muscles from $Cx43^{fl/fl}$:MC (light blue), $Cx45^{fl/fl}$:MC (orange), $Panx1^{-/-}$ (fuchsia), and $P2X_7^{-/-}$ (burgundy) mice were used to determine Etd^+ uptake at 7 days post-denervation (**a** and **b**) or at 48 h of culture (**c** and **d**). After 10 min of baseline recording, 200 µM La^{3+} was added. N=5; at least six myofibers recorded in each animal, each value is the mean ± SEM. Discontinuous line represents the average for $Cx43^{fl/fl}Cx45^{fl/fl}$ mice, dotted line represents the average $Cx43^{fl/fl}Cx45^{fl/fl}$: MC mice. *** $p < 0.001$, for denervated myofibers compared with innervated myofibers. ### $p < 0.001$, for effect of La^{3+} compared with basal conditions by ANOVA with Bonferroni *post hoc* test.

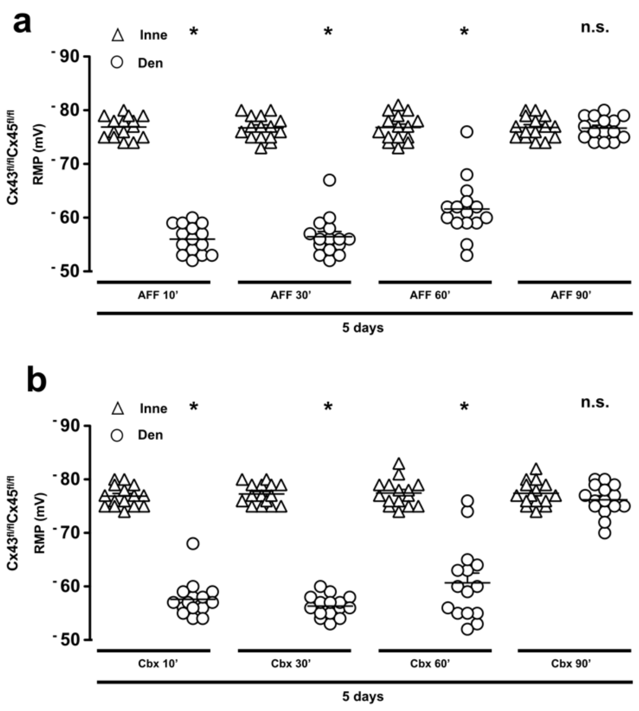

Supplementary Figure 12. Inhibition of connexin hemichannels reverts the decrease in resting membrane potential in denervated skeletal myofibers. Unilateral sciatic nerve transections were performed in $Cx43^{fl/fl}Cx45^{fl/fl}$ mice and *in vivo* resting membrane potential (RMP) was measured in myofibers of *flexor digitorum brevis muscle* of both limbs at 5 days post-denervation (innervated: Ine [∆], denervated: Den [○]). In **a**, the measurements were first performed in Krebs-BTS-HEPES solution (recording medium), then in **b**, the presence of 300 µM flufenamic acid (AFF) or 100 µM carbenoxolone (Cbx), respectively, at 10, 30, 60 and 90 min of incubation. N=5; at least twenty myofibers recorded in each animal. n.s.: non-significant difference; * $p < 0.05$, for denervated myofibers compared with innervated myofibers by ANOVA with Bonferroni *post hoc* test.

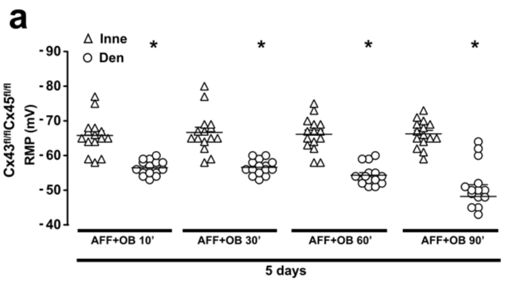

Supplementary Figure 13. The decrease in resting membrane potential (RMP) of denervated skeletal myofibers is not due to malfunction of the Na^+/K^+ ATPase pump. Unilateral sciatic nerve transections were performed in $Cx43^{fl/fl}Cx45^{fl/fl}$ mice. At 5 d post-denervation, RMP was measured *in vivo* in myofibers of *flexor digitorum brevis* muscle from both limbs. Measurement was performed first in Krebs-BTS-HEPES solution (recording medium), then in the presence of 300 µM flufenamic acid and 300 nM ouabain (AFF + OB) at 10, 30, 60 and 90 min of incubation. N=5; at least twenty myofibers were recorded in each animal. * $p < 0.05$, for denervated myofibers compared with innervated myofibers by ANOVA with Bonferroni *post hoc* test.

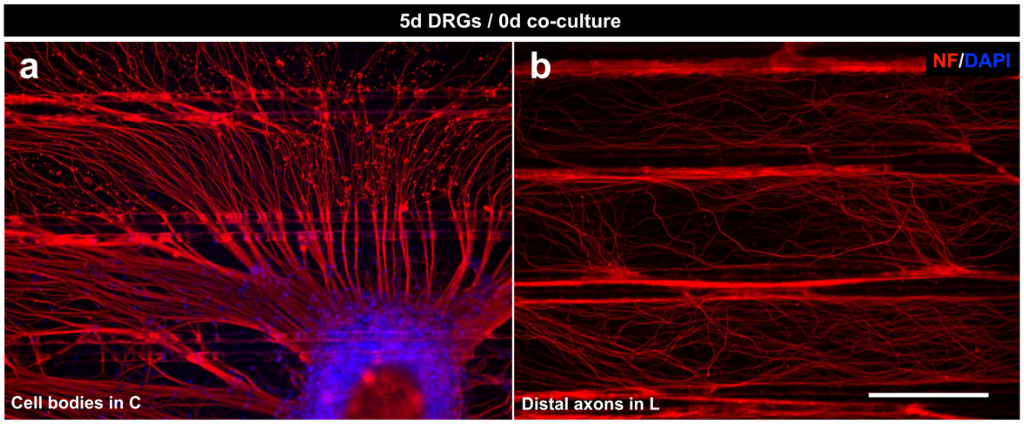

Supplementary Figure 14. Dorsal root ganglion (DRG) cultures at 5 days. Culture of DRGs in Campenot chambers 5 days. In **a**, Cell bodies of neurons, and Schwann cells, or any other cells extracted together with the DRG were observed in the central chamber (C). In **b**, distal neurites without cell bodies are seen in lateral (L) chambers, to which they were guided by superficial scratches. Immunofluorescence using antibodies against neurofilament 200 (NF; red), and DAPI staining (blue) at 0 days of co-culture. Scale bar: 100 µm.

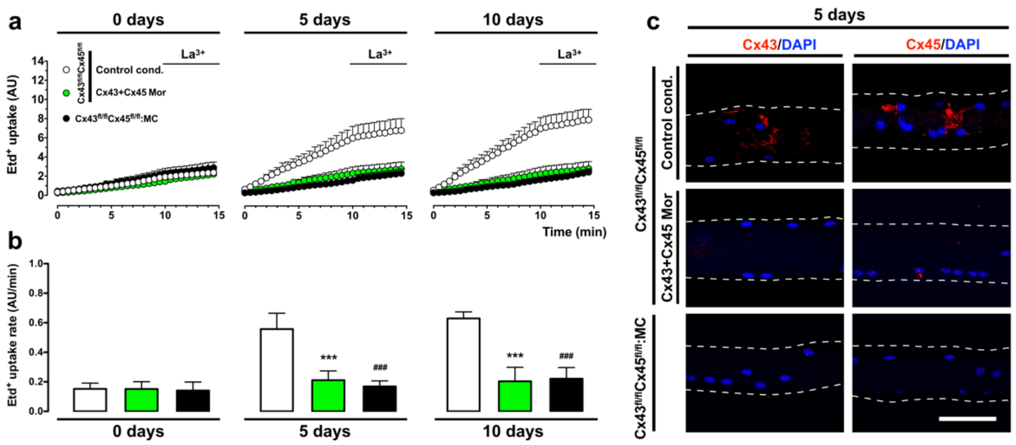

Supplementary Figure 15. Functional Cx HCs are expressed in cultured myofibers at 5 and 10 days of culture. In **a**, Time-lapse of ethidium (Etd^+) uptake in skeletal myofibers from $Cx43^{fl/fl}Cx45^{fl/fl}$ mice in control conditions (Control cond.; white), or from $Cx43^{fl/fl}Cx45^{fl/fl}$ mice in presence of morpholinos to Cx43 and Cx45 (Cx43+Cx45 Mor; green), or from $Cx43^{fl/fl}Cx45^{fl/fl}$:MC mice (black) at 0, 5 or 10 days of culture. The first 10 min of recordings are baseline; the next 5 min were in the presence of 200 μM La^{3+}. In **b**, Etd^+ uptake rates from recordings in a. N=5; at least seven myofibers recorded in each independent experiment, each value is the mean ± SEM. *** $p < 0.001$, for Cx43+Cx45 Mor compared with control conditions. ### $p < 0.001$, for $43^{fl/fl}Cx45^{fl/fl}$:MC mice compared with control conditions by two-way ANOVA with a Bonferroni post hoc test. In **c**, Immunofluorescence using antibodies against Cx43 or Cx45 for myofibers from $Cx43^{fl/fl}Cx45^{fl/fl}$ mice in Control cond. (top row), or in the presence of Cx43+Cx45 Mor (middle row), and $Cx43^{fl/fl}Cx45^{fl/fl}$:MC mice (bottom row) at 5 days of culture using antibodies against Cx43 (left column), or Cx45 (right column), and DAPI staining (blue). The dotted line is the edge of the myofibers. Scale bar: 50 μm.
